# Comparative analysis of crab growth performance, enzyme activity, and microbiota between rice-crab coculture and pond farming systems

**DOI:** 10.3389/fvets.2025.1571454

**Published:** 2025-03-19

**Authors:** Xiaochen Zhu, Miao Nie, Na Sun, Yazhao Zhang, Mingxia Sun, Changlei Li, Qing Jiang, Hua Wei, Yingdong Li, Qingbiao Hu, Yingying Zhao, Xiaodong Li

**Affiliations:** ^1^Hebei Key Laboratory of the Bohai Sea Fish Germplasm Resources Conservation and Utilization, Beidaihe Central Experiment Station, Chinese Academy of Fishery Sciences, Qinhuangdao, China; ^2^College of Animal Science and Veterinary Medicine, Shenyang Agricultural University, Shenyang, China; ^3^College of Science and Engineering, Flinders University, Bedford Park, SA, Australia; ^4^Panjin Guanghe Crab Industry Co. Ltd., Panjin, China; ^5^Key Laboratory of Breeding and Propagation of Chinese Mitten Crab, Ministry of Agriculture and Rural Affairs, Panjin, Liaoning, China; ^6^Liaoning Panjin Wetland Ecosystem National Observation and Research Station, Shenyang, China

**Keywords:** *Eriocheir sinensis*, gut microbiota, environmental microbiota, culture system, *16S rRNA*, growth performance, enzyme activity

## Abstract

**Introduction:**

To support the sustainable development of rice and aquaculture industries, various rice-animal coculture systems have been developed. One such system, the rice-crab coculture system (RCC), has been practiced for decades in northern China. However, studies on the crab physiological status in RCC remain limited. Microorganisms play a crucial role in aquaculture by influencing animal nutrition, health, nutrient cycling, water quality, and environmental impact. Research on the gut and environmental microbiota in RCC is scarce.

**Methods:**

This study compared the growth performance, immune and digestive enzyme activities of crabs between RCC and traditional pond farming system (PF). In addition, the microbiota in crab guts, water, and sediment from both systems was investigated using *16S rRNA* gene sequencing.

**Results:**

Crabs in RCC exhibited superior growth performance and higher enzymatic activities, including acid phosphatase (ACP), alkaline phosphatase (AKP), lipase (LPS), and trypsin (TRY). Significant differences were observed in microbiota composition across crab gut, water, and sediment samples, respectively. RCC crabs had a lower abundance of Bacteroidota and a higher abundance of Firmicutes in their gut microbiota. The RCC environment was enriched with beneficial bacteria such as Rhizobiales, Methylococcales, KD4-96, C39, Xanthomonadales, and Nitrosomonadaceae. Microbial function predictions confirmed enhanced methanotrophy and nitrogen fixation in the RCC.

**Discussion:**

The RCC enhances the growth rate and immune capability of crabs. Crabs from RCC consume more animal-based nutrition, which results in distinct differences in gut microbiota composition and higher levels of LPS and TRY compared to those in PF. Additionally, RCC supports environmentally beneficial bacteria that contribute to greenhouse gas reduction, carbon and nitrogen fixation, organic matter decomposition, and ammonia oxidation, benefiting both the crabs and their ecosystem. These findings enhance our understanding of crab physiology and microbial communities in RCC and PF systems.

## 1 Introduction

The Chinese mitten crab (*Eriocheir sinensis*) is one of the most important freshwater crustaceans in China due to its commercial and ecological value (1, 2). Chinese mitten crab cultivation is practiced widespread across China (3), with annual production reaching 7.76 million tons in 2020 (4). The primary adult crab culture methods of Chinese mitten crabs include traditional pond farming system (PF) and rice-crab coculture system (RCC) (3). The former can cause severe environmental issues, such as eutrophication, greenhouse gas emissions, and acidification (5, 6). In contrast, RCC is considered a more sustainable method (7). Similar to other rice-animal coculture systems, RCC is not only more profitable than rice monoculture systems but also reduces the use of pesticides and fertilizers (8). Consequently, the Chinese government has promoted this culture system for many years (3). In 2019, China's rice-crab coculture area was ~138,600 hm^2^, and the annual yield reached 61,800 tons (9, 10). Previous research has investigated the effect of stocking density on the yield and nutritional quality of crabs in the RCC, suggesting that an appropriate density can significantly affect crab growth rate, gonadosomatic index, and meat yield (11–13). The positive ecological impact of RCC has also been confirmed in some studies. For example, with the same fertilizer N application, RCC improved rice yield compared to rice monoculture (RM) and also increased crab yield compared to crab monoculture when provided the same amount of feed (14). Moreover, RCC enhances nutrient levels in the soil and rice compared to RM (15). Additionally, through optimizing crab density and surplus N from crab feed, RCC can significantly reduce the emission of methane (CH_4_) and nitrous oxides (N_2_O), which are potent greenhouse gases (16). However, studies on the physiological status of crabs are limited.

Microorganisms play an essential role in aquaculture systems. They are closely related to the nutrition and health status of the culture animals and the productivity, nutrient cycling, water quality, and environmental impact of the effluent (17, 18). As one of the most significant aquaculture species, numerous comparative studies on the gut microbiota of Chinese mitten crabs, focusing on factors such as genetic background, developmental stage, habitat, feedstuff, health status, and environmental conditions, have been reported (19–25). However, there are very limited studies on environmental microorganisms in Chinese mitten crab culture ecosystems (23, 26), especially in RCC.

This study aimed to compare the growth performance, immune and digestive enzyme activities, and gut microbiota of crabs cultured in PF and RCC systems. Additionally, we analyzed environmental microorganisms in water and sediment to assess the differences in these systems. These findings expand our understanding of the physiological status of crabs and microbial communities in ecosystems in PF and RCC systems.

## 2 Materials and methods

### 2.1 Experimental design

The experiments were conducted in Panjin Guanghe Crab Industry Co., Ltd. in Panjin City, China (N 40°54′, E121°52′). The rice field preparation followed the methods of Li et al. (11). Prior to the experiment, the juvenile crabs were kept in the wintering ponds. Two weeks after rice seedlings were transplanted, the juvenile crabs pooled from wintering ponds with an average weight (mean ± SD) of 10 ± 0.2 g were transferred to the experimental ponds (three replicates, approximate size 0.1 ha) and rice fields (three replicates, approximate size 0.15 ha) at a density of 2500 crabs per pond or rice field in June 1st, 2021. Before being transferred to the plots, the robust crabs (showing no disease and visually assessed healthy intestine and hepatopancreas) were randomly collected from pooled juvenile crabs. Water and sediment samples were collected from three experimental ponds and rice fields, respectively (Table 1).

**Table 1:** Sample collection information.

**Sampling time**	**Culture system**	**Sample type**	**Sample name**	**Sample No**.	**BioProject No. (Genbank)**
June 2021	NA	Juvenile crab gut	CGJ	18	PRJNA1142732 (SAMN42956269-SAMN42956286)
	PF	Water	PWJ	3	PRJNA1142913 (SAMN42972939-SAMN42972941)
		Sediment	PSJ	3	PRJNA1144156 (SAMN43027899-SAMN43027901)
	RCC	Water	RWJ	3	PRJNA1142913 (SAMN42972945-SAMN42972947)
		Sediment	RSJ	3	PRJNA1144156 (SAMN43027905-SAMN43027907)
September 2021	PF	Adult crab gut	PGS	18	PRJNA1142732 (SAMN42956251-SAMN42956268)
		Water	PWS	3	PRJNA1142913 (SAMN42972942-SAMN42972944)
		Sediment	PSS	3	PRJNA1144156 (SAMN43027902-SAMN43027904)
	RCC	Adult crab gut	RGS	18	PRJNA1142732 (SAMN42956287- SAMN42956304)
		Water	RWS	3	PRJNA1142913 (SAMN42972948-SAMN42972950)
		Sediment	RSS	3	PRJNA1144156 (SAMN43027908-SAMN43027910)

The management of the two culture systems was identical. During the experimental period, crabs were fed twice daily with a commercial pelleted feed (Wellhope Aquatic Feed Co. Ltd., Shenyang, China). The feeding amount was adjusted by 5%–10% based on the size and population of the crabs. Water was added when the water level in the pond or rice field was significantly reduced, with no drainage occurring during the experiment.

Crabs from both culture systems were harvested in September 1st, 2021. After the harvest, robust crabs, water, and sediment were collected from each experimental pond and rice field for further analysis (Table 1). 100 crabs from each pond and rice field were randomly collected to measure their carapace length, width, height and weight to evaluate their growth performances followed the methods described by Xue et al. (27).

### 2.2 Sample collection

#### 2.2.1 Gut and hepatopancreas samples

The crabs were temporarily raised in the same tank under continuous aeration at ~20°C in the lab. After 24-h fast, six individuals were randomly collected from each group. We rinsed the crab's shell surfaces with sterile water and wipe the surfaces using 75% ethanol. The gut and hepatopancreas were dissected immediately in a sterile environment. Approximately 1.5 cm of mid and hindgut from each individual crab was collected as one replicate for gut microbiota sequencing. Meanwhile, three individual hepatopancreas samples were pooled as one replicate for enzyme activity assessment. Both gut and hepatopancreas replicates were placed in 2 mL cryogenic vials, submerged in liquid nitrogen, and stored at −80°C for subsequent analyses.

#### 2.2.2 Water and sediment samples

In each experimental pond and rice field, water and sediment samples were collected from six spots (four corners and two middle spots of the long sides) and then pooled as one biological replicate. Water samples were collected from 5 cm below the water surface using sterile bottles. In each pond or rice field, 500 mL of water from each sample was filtered through a 0.22 μm polycarbonate membrane filter (Millipore, Germany). The filters were then stored at −80°C in 2 mL cryogenic vials for DNA extraction. Sediment samples were taken using plastic pipes (diameter 2.5 cm), and filtered through a 2 mm filter to remove large debris. At last, ~2 g sediment for each pond or rice field was placed into a 2 mL cryogenic vial at −80°C for DNA extraction.

### 2.3 Analysis of enzyme activities

Three hepatopancreas replicates (each replicate pooled from the hepatopancreas of three crabs) tissue was used for enzyme activity assessment. The assessment included two immune-related enzymes [acid phosphatase (ACP) and alkaline phosphatase (AKP)] and three digestive enzymes [amylase (AMS), lipase (LPS), and trypsin (TRY)] by using assay kits (Nanjing Jiancheng Bioengineering Institute, China) according to the manufacturer's protocols. Detailed methods and equipment for assessing the above enzymes and total protein content (TP) are shown in Supplementary Table S1.

### 2.4 Microbiota sequencing

Microbial community genomic DNA of crab gut, water and sediment was extracted with an E.Z.N.A.^®^ soil DNA Kit (Omega Bio-tek, Norcross, GA, USA) following the manufacturer's instructions. DNA concentration and purity were measured by NanoDrop 2000 (Thermo Scientific, Wilmington, USA). The hypervariable region V3-V4 of the bacterial *16S rRNA* gene was amplified with primer pairs 338F (5′-ACTCCTACGGGAGGCAGCAG-3′) and 806R (5′-GACTACHVGGGTWTCTAAT-3′). The PCR reaction mixture included 4 μL 5 × FastPfu buffer, 2 μL 2.5 mM dNTPs, 0.8 μL each primer (5 μM), 0.4 μL Fast Pfu polymerase, 10 ng of template DNA, and ddH_2_O to a final volume of 20 μL. PCR amplification cycling conditions for gut samples were as follows: initial denaturation at 95°C for 3 min, followed by 29 cycles of denaturation at 95°C for 30 s, annealing at 53°C for 30 s, and extension at 72°C for 45 s, and single extension at 72°C for 10 min, and end at 4°C. For water and sediment samples, the annealing temperature was 55°C and the number of PCR cycles was 27, while other conditions were the same for gut samples. The PCR product was extracted from 2% agarose gel and purified using the PCR Clean-Up Kit (YuHua, Shanghai, China) according to manufacturer's instructions and quantified using Qubit 4.0 (Thermo Fisher Scientific, USA). Purified amplicons were pooled in equimolar and paired-end sequenced on an Illumina MiSeq PE300 platform (Illumina, San Diego, USA) under the standard protocols by Majorbio Bio-Pharm Technology Co. Ltd. (Shanghai, China). The raw reads were deposited into the NCBI Sequence Read Archive (SRA) database under the BioProject number PRJNA1142732, PRJNA1142913, and PRJNA1144156 (Table 1).

### 2.5 Data processing and statistical analyses

After sequencing, the raw *16S rRNA* gene sequencing reads were demultiplexed and quality-filtered by fastp version 0.20.0 (28). The reads were merged by FLASH 1.2.11 (29) with the same criteria as described in a previous study (30). Operational taxonomic units (OTUs) were clustered using UPARSE version 7.1 (31) with a 97% similarity cutoff, and chimeric sequences were identified and removed. The taxonomy of each OTU representative sequence was analyzed by RDP Classifier version 2.2 (32) against the *16S rRNA* database (Silva SSU 138) using a confidence threshold of 0.7. After removing chloroplast and mitochondria sequences, the Chao and Shannon indices for α-diversity were calculated by Mothur 1.30.2 (33). β-diversity was determined through Bray-Curtis dissimilarity matrices and displayed using principal coordinate analysis (PCoA). To identify differences between groups, we conducted an Analysis of similarities (ANOSIM) using Bray-Curtis dissimilarity. A linear discriminant analysis (LDA) effect size (LEfSe) was performed to present the enrichment of microbial communities within the different samples (34). Functional profiling was inferred from the relative abundance of *16S rRNA* sequences using Phylogenetic Investigation of Communities by Reconstruction of Unobserved States (PICRUSt2) (35). Additionally, the Functional Annotation of Prokaryotic Taxa (FAPROTAX) (36) was used to predict the ecological relevant functions of bacterial community in water and sedimental samples.

All of these analyses were carried out on the Majorbio I-Sanger Cloud Platform (37). One-way ANOVA with Waller-Duncan test was performed using the SPSS 20.0 software package (SPSS Inc., Chicago, USA) to estimate the differences of Shannon and Chao indices within different sample types or ecosystems, and hepatopancreas enzymatic activity level among crabs in June (CJ), crabs in September from PF (PS) and RCC (RS). Significance was declared at *P* < 0.05. Additionally, One-way ANOVA was employed to assess differences in relative abundance in KEGG pathways (Level 3) and ecological functions within gut, water, and sediment samples. *P*-values were corrected using the False Discovery Rate (FDR) method, with significance was set at corrected *P-*values < 0.05.

## 3 Results

### 3.1 Growth performance and enzymatic activities in Chinese mitten crabs

The growth parameters are shown in Table 2. Crabs from RCC were significantly higher in all parameters compared to crabs from PF, indicating they have a better growth performance.

**Table 2:** Growth performance of Chinese mitten crabs between PF and RCC system.

**Parameter**	**Culture system**	
	**PF**	**RCC**
Weight (g)	77.6 ± 21.1^a^	97.5 ± 27.5^b^
Shell length (cm)	50.9 ± 4.4^a^	55.4 ± 5.0^b^
Shell width (cm)	55.2 ± 4.7^a^	58.9 ± 5.2^b^
Shell Height (cm)	28.2 ± 2.9^a^	30.2 ± 2.9^b^

Different letters indicate significant difference between PF and RCC systems.

**Figure 1:**
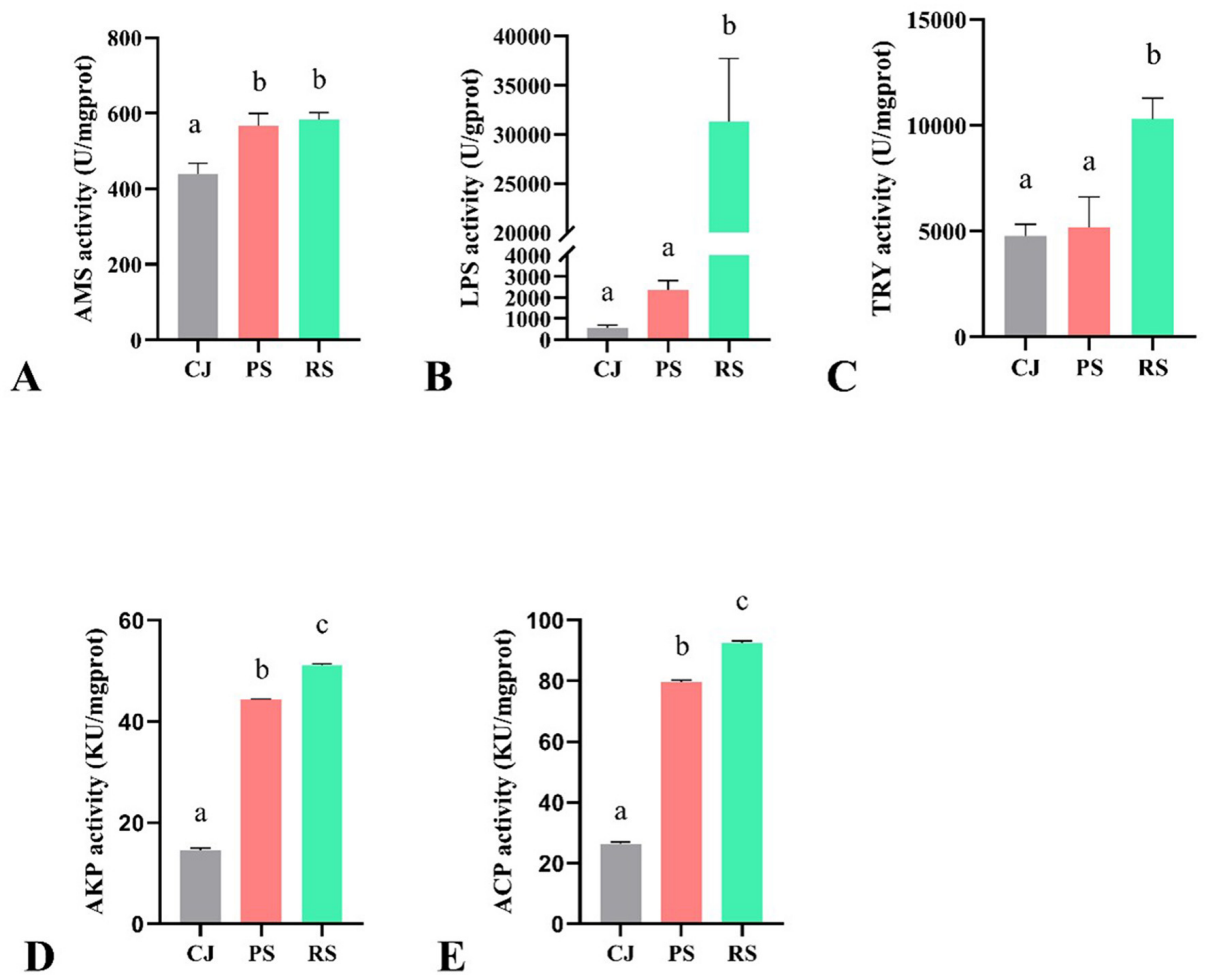
Enzyme activity of Chinese mitten crabs under two culture systems. **(A)** AMS; **(B)** LPS; **(C)** TRY; **(D)**: AKP; **(E)** ACP. CJ, crabs in June; PS, crabs under PF in September; RS, crabs under RCC in September; KU, King's unit. Different *lowercase letters* indicate significant differences (*P* < 0.05).

The enzyme activities among crabs in June (CJ), crabs in September from PF (PS) and RCC (RS) are shown in Figure 1. The amylase (AMS) activity of crabs under both culture systems in September (PS and RS) did not differ significantly, but they were significantly higher than those of the CJ group. Lipase (LPS) and trypsin (TRY) activities in RS were significantly higher than in CJ and PS, while the latter two showed no significant difference. Acid phosphatase (ACP) and alkaline phosphatase (AKP) activities in CJ was significantly lower than in PS and RS, whereas in RS, they were significantly higher than in PS.

### 3.2 Overview of *16s rRNA* sequencing data

A total of 1,501,890 OTUs were obtained, with an average of 56,976, 46,197, and 57,184 OTUs in the gut, water, and sediment microbial communities, respectively. The α-diversity indices, including the Chao and Shannon indices, were calculated from the OTUs of each group (Table 3), the group abbreviations are explained in Table 1.

**Table 3:** α-diversity indices of gut, water and sediment samples from two different culture systems of Chinese mitten crabs.

**Sample type**	**Group**	**Shannon**	**Chao**
**Gut**	CGJ	2.65 ± 0.18	312.01 ± 47.64^a^
	PGS	2.95 ± 0.13	624.4 ± 44.12^b^
	RGS	2.7 9± 0.14	395.01 ± 52.21^a^
**Water**	PWJ	3.34 ± 0.03^a^	473.11 ± 38.03^a^
	RWJ	3.96 ± 0.13^b^	1252.87 ± 139.76^bc^
	PWS	3.99 ± 0.23^b^	1127.03 ± 190.36^b^
	RWS	4.65 ± 0.02^c^	1669.82 ± 61.28^c^
**Sediment**	PSJ	6.44 ± 0.08^a^	3997.98 ± 156.8^ab^
	RSJ	6.71 ± 0.02^ab^	3792.63 ± 99.19^a^
	PSS	6.62 ± 0.12^ab^	4397.89 ± 170.3^b^
	RSS	6.84 ± 0.07^b^	4200.9 ± 92.58^ab^

Lowercase letters indicate significant differences in the same column within same sample types (*P* < 0.05).

In the gut microbial community, there were no significant differences in the Shannon index among these three groups, although the values of PGS and RGS were higher than for CGJ. The Chao index was the highest in PGS (624.40 ± 44.12), which was significantly higher than those observed in CGJ (312.01 ± 47.64) and RGS (395.01 ± 52.21).

Regarding the water microbial community, compared to PF, RCC had higher Shannon and Chao indices in June and September, respectively. The Shannon indices significantly increased in both PF [3.34 ± 0.03 (PWJ) to 3.99 ± 0.23 (PWS)] and RCC [3.96 ± 0.13 (RWJ) to 4.65 ± 0.02 (RWS)]. A similar pattern was also observed in Chao indices in the water microbial community.

Concerning the sediment microbial community, Shannon and Chao indices were also higher in September than in June in both systems, but the increases were not statistically significant. At two sampling times, higher Shannon indices were observed in RCC [6.71 ± 0.02 (RSJ) vs. 6.44 ± 0.08 (PSJ), 6.84 ± 0.07 (RSS) vs. 6.71 ± 0.02 (PSS), while higher Chao indices were observed in PF [3997.98 ± 156.8 (PSJ) vs. 3792.63 ± 99.19 (RSJ), 4397.89 ± 170.3 (PSS) vs. 4200.9 ± 92.58 (RSS)], although the differences were not significant.

**Figure 2:**
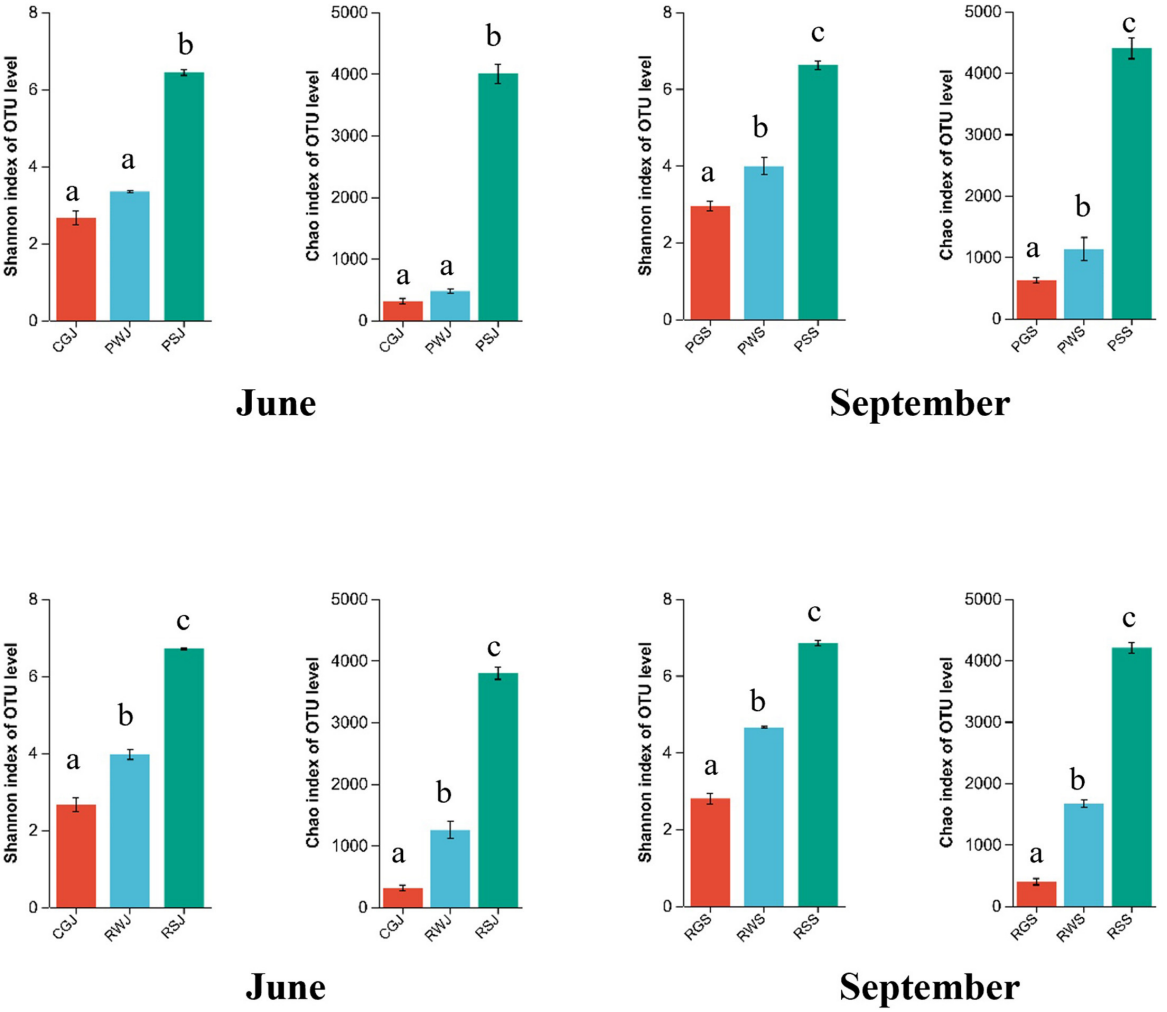
Shannon and Chao indices of gut, water, and sediment within the same ecosystem. *Lowercase letters* indicate significant difference (*P* < 0.05).

In the comparison of the α-diversity of gut, water, and sediment microbial communities in each ecosystem, the Shannon and Chao indices were significantly higher in the sediment, followed by the water, with the gut having the lowest indices. Except the PF in June, the Shannon and Chao indices of water were significantly higher than those of the gut (Figure 2).

### 3.3 Differences in microbiota between the two culture systems

**Figure 3:**
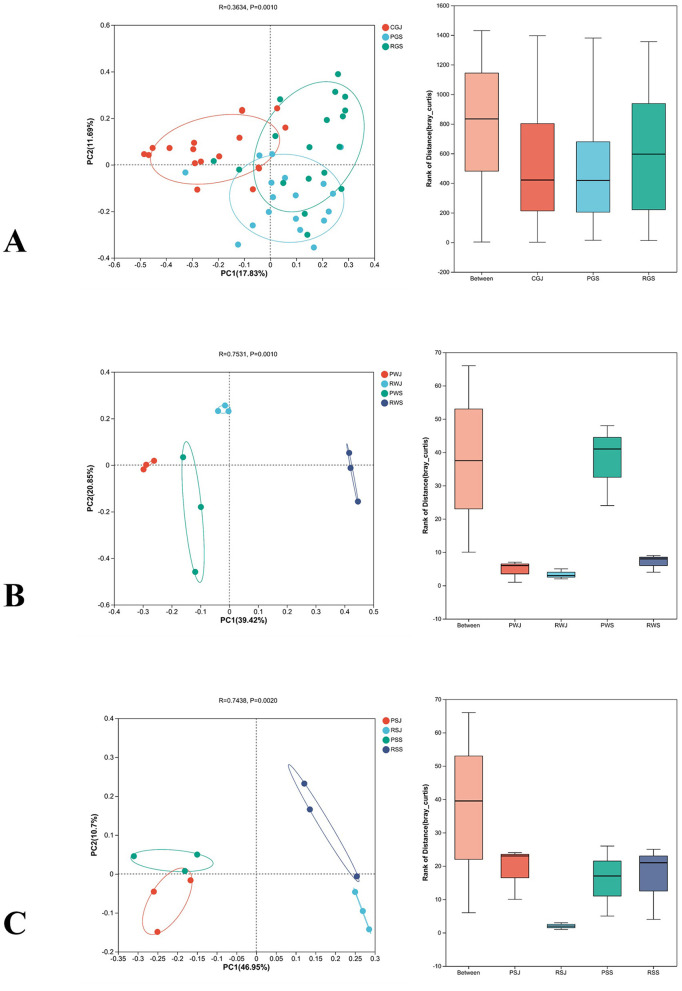
β-diversity comparison of the gut **(A)**, water **(B)** and sediment microbiota **(C)** between two culture systems in June and September at the OTU level.

Differences in bacterial community compositions of intestines, water, and sediment between two culture systems in different months were visualized by conducting the PCoA based on Bray-Curtis distances. ANOSIM analysis and β-diversity analysis through PCoA indicated significant differences in gut microbiota (*R* = 0.3634, *P* = 0.001; Figure 3A), water microbiota (*R* = 0.7531, *P* = 0.001; Figure 3B), and sediment microbiota (*R* = 0.7438, *P* = 0.002; Figure 3C).

### 3.4 Microbiota compositions in the two culture systems

**Figure 4:**
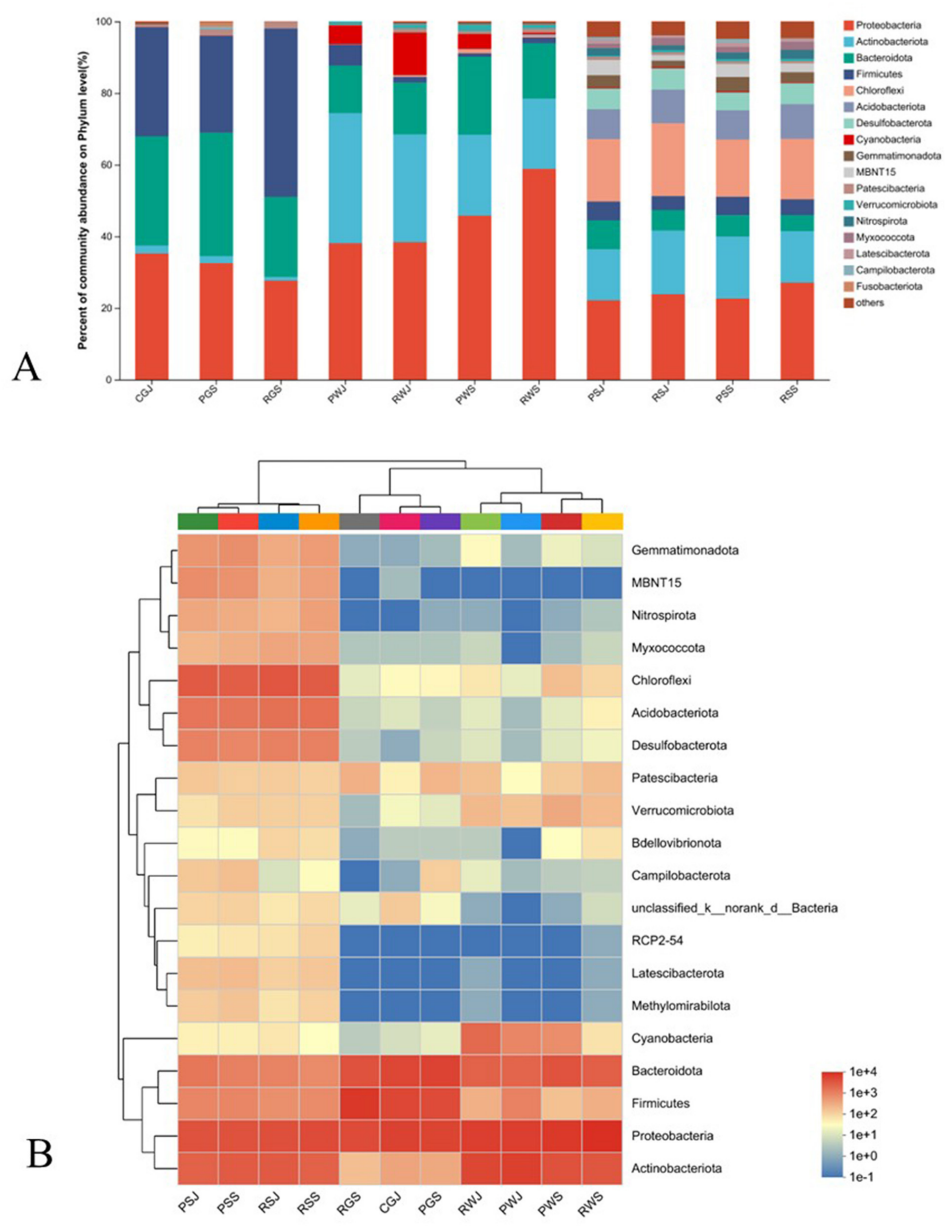
Gut, water and sedimental microbiota composition **(A)** and heatmap **(B)** at the phylum level.

The relative abundance of bacterial community in different samples at the phylum level is shown in Figure 4A. The community heatmap suggests apparent divergence among gut, water, and sediment samples (Figure 4B).

**Figure 5:**
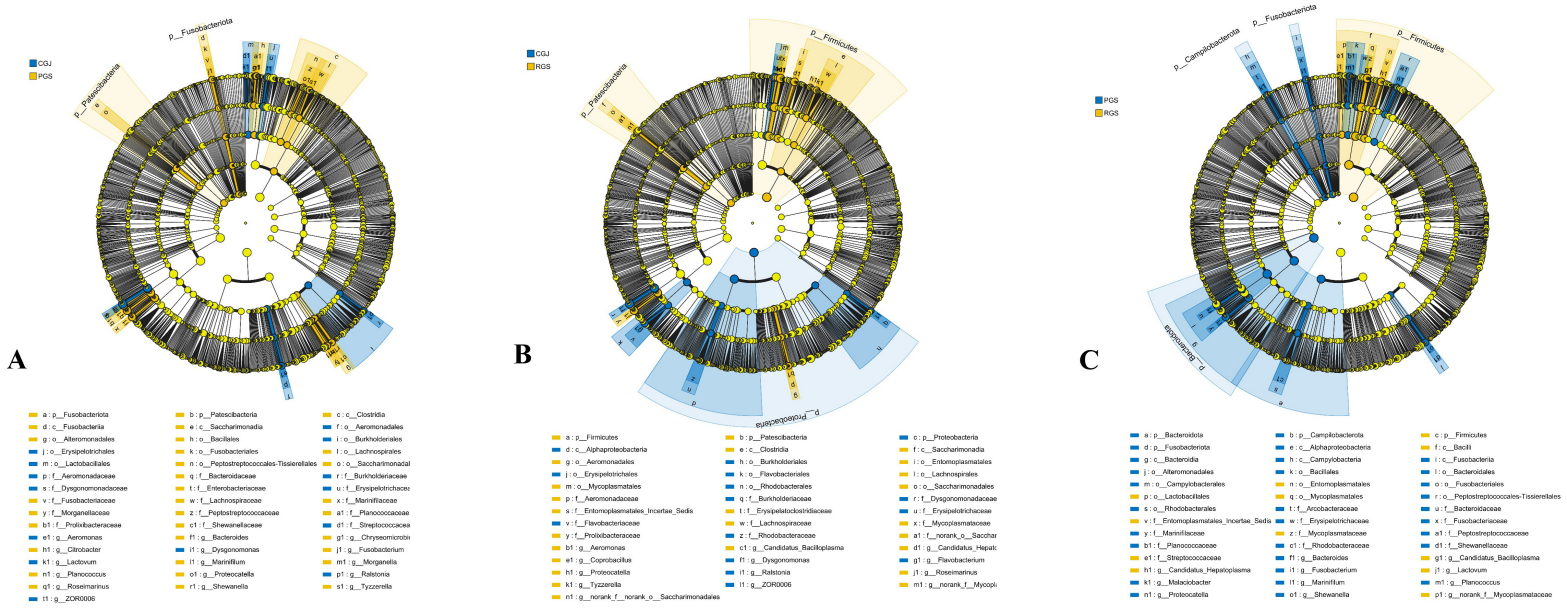
The linear discriminant analysis EffectSize (LEfSe) displaying the differences in the gut microbiota between CGJ and PGS **(A)**, CGJ and RGS **(B)**, and PGS and RGS **(C)**. LDA score > 3.5.

In the crab gut, Proteobacteria (27.64%–35.24%), Bacteroidota (22.34%–34.49%), and Firmicutes (27.07%–47.04%) were the three most dominant phyla in all three gut groups. Figure 5 shows the microorganisms with significant differential abundance between CGJ and PGS, CGJ and RGS, and PGS and RGS. Compared with CGJ, PGS had a higher abundance in two phyla (Patescibacteria and Fusobacteriota), three classes (Saccharimonadia, Clostridia, and Fusobacterila), six orders, ten families and 11 genera. CGJ was richer in four orders (Aeromonadales, Burkholderiales, Erysipelotrichales, and Lactobacillales), five families, and five genera. Between CGJ and RGS, RGS had a significantly higher abundance in two phyla (Firmicutes and Patescibacteria), two classes (Saccharimonadia and Clostridia), five orders, seven families and nine genera. CGJ had a higher abundance in the one phylum (Proteobacteria), one class (Alphaproteobacteria), five orders, six families, and six genera. In the comparison between PGS and RGS, PGS was rich in three phyla (Bacteroidota, Fusobacteriota, and Campilobacterota), four classes, eight orders, ten families, and eight genera, but lower in Firmicutes (phylum level), Bacilli (class level), Entomoplasmatales, Lactobacillales, and Mycoplasmatales (order level), three families, and four genera.

**Figure 6:**
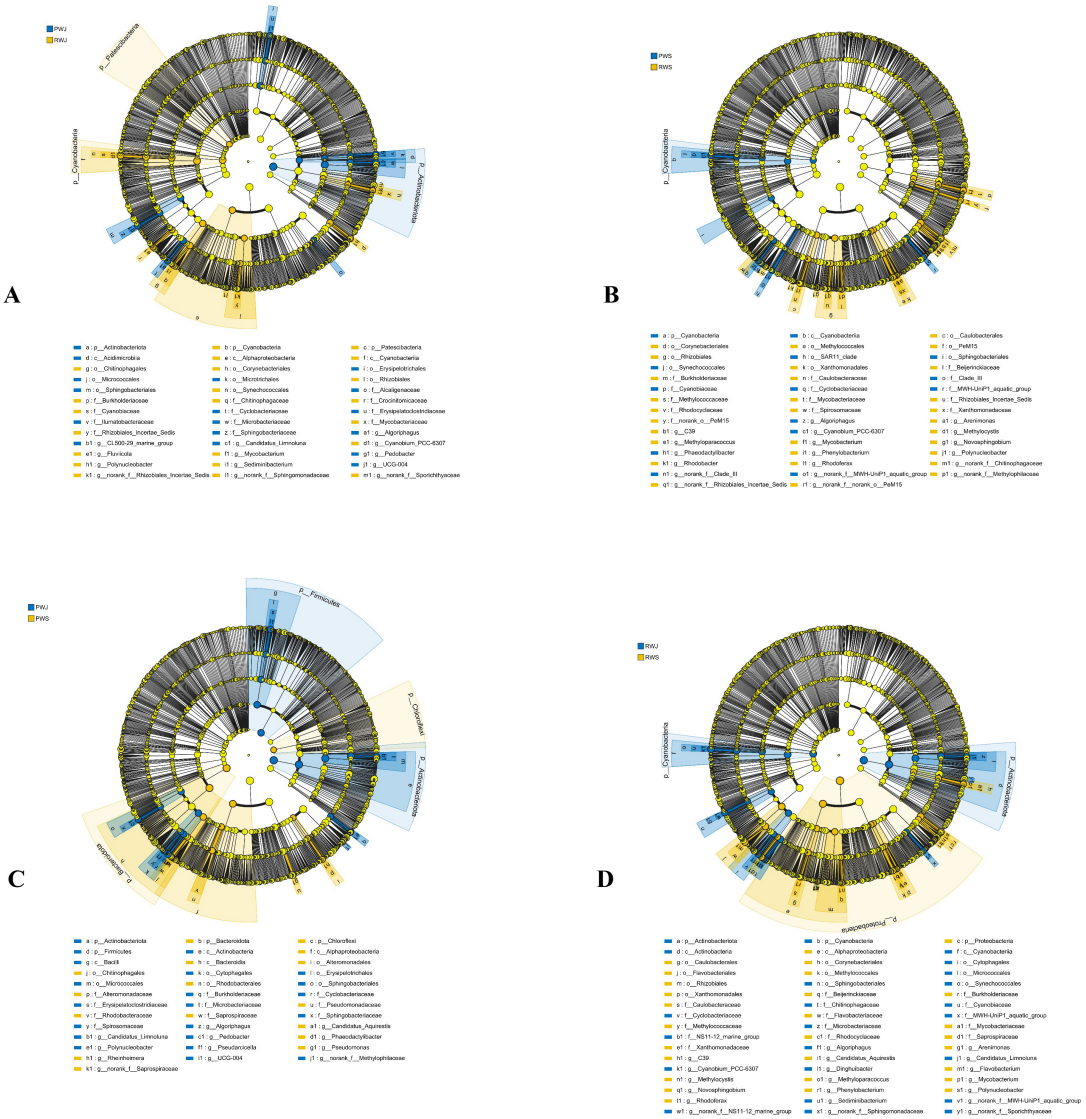
The linear discriminant analysis EffectSize (LEfSe) displaying the differences in the water between PWJ and RWJ **(A)**, PWS and RWS **(B)**, PWJ and PWS **(C)**, and RWJ and RWS **(D)**. LDA score > 3.5.

Regarding the water microbial community, Proteobacteria (38.06%–58.84%), Actinobacteriota (19.60%–36.33%), Bacteroidota (13.28%–21.72%), and Cyanobacteria (0.39%–11.82%) were the four most dominant phyla in all samples (Figure 4). In June, PWJ had a higher abundance of Actinobacteriota but a lower abundance of Patescibacteria and Cyanobacteria compared with RWJ (Figure 6A). In the comparison between PWS an RWS, PWS was rich in Cyanobacteria (phylum level), Cyanobacteriia (class level), Synechococcales, Sphingobacteriales, SAR11_clade, and unclassified_Actinobacteria (order level), while RWS was rich in Rhizobiales, Caulobacterales, Corynebacteriales, Methylococcales, Xanthomonadales, and Pem15 (order level; Figure 6B). In the PF, a higher abundance of Bacteroidota and Chloroflexi were observed in PWS, while a higher abundance of Firmicutes and Actinobacteriota were found in PWJ (Figure 6C). In the RCC, compared with RWJ, RWS had a higher abundance of Proteobacteria but a lower abundance of Actinobacteriota and Cyanobacteria (Figure 6D).

**Figure 7:**
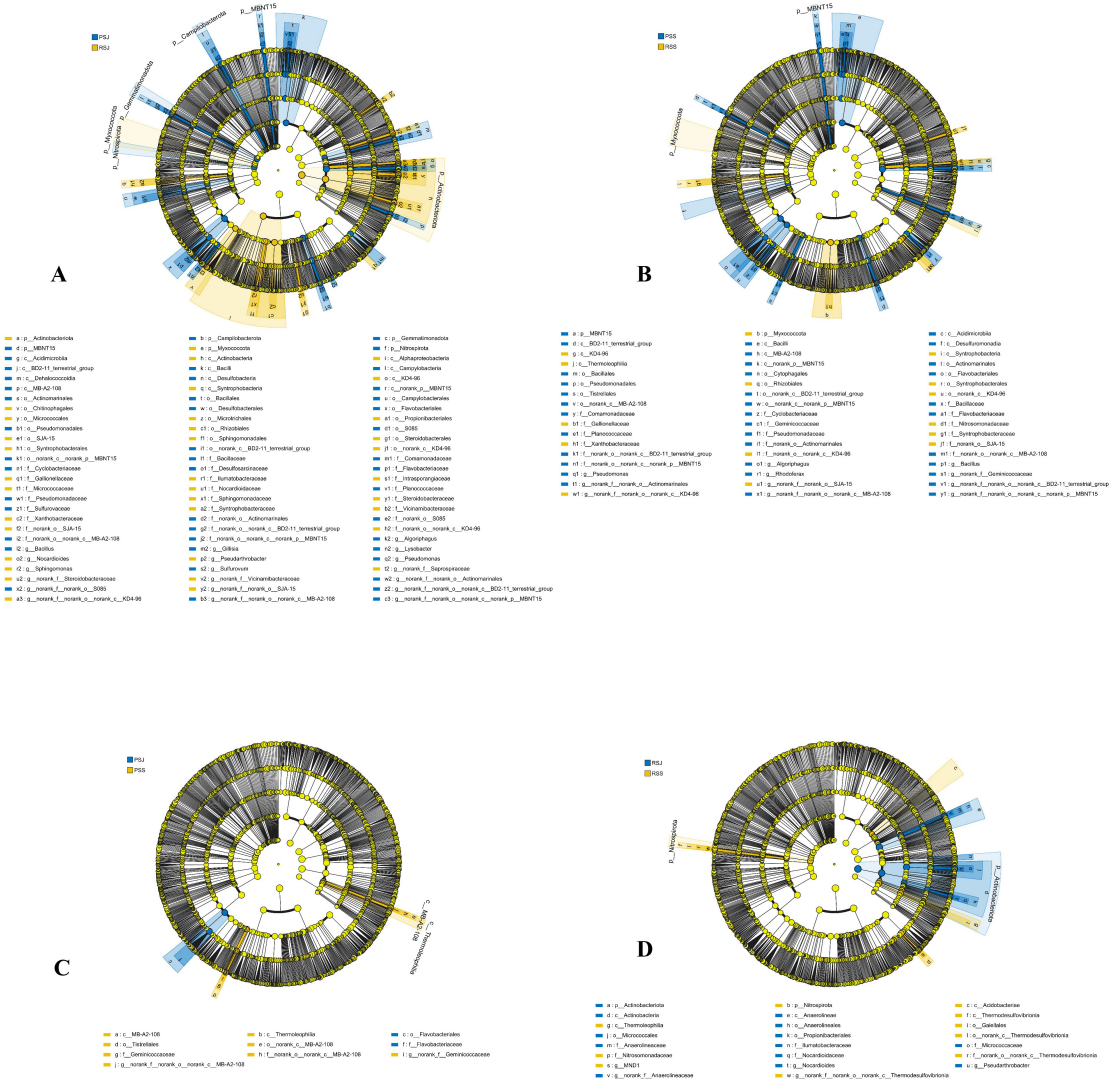
The linear discriminant analysis EffectSize (LEfSe) displaying the differences in the sediment between PSJ and RSJ **(A)**, PSS and RSS **(B)**, PSJ and PSS **(C)**, and RSJ and RSS **(D)**, LDA score > 3.5.

In the sediment samples, Proteobacteria (22.14%–27.07%), Chloroflexi (15.94%–20.34%), Actinobacteriota (14.29%–17.78%), and Acidobacteriota (8.16%–9.62%) were the most dominant phyla. Bacteroidota, Desulfobacterota, and Firmicutes also had higher relative abundance (3.89%–8.03%) than those low-abundance phyla (Figure 4A). In June, RSJ was rich in phyla of Actinobacteriota and Myxococcota, while PSJ was rich in phyla of MBNT15, Nitrospirota, Campilobacterota, and Gemmatimonadota (Figure 7A). Compared with RSS, PSS was rich in MBNT15 (phylum level), Bacilli, Acidimicrobiia, Desulfuromonadia, MB-A2-108, and BD2-11 terrestrial group (class level), and eight orders, whilst RSS was rich in KD4-96, Thermoleophilia and Syntrophobacteria (class level), Rhizobiales, Syntrophobacterales, and unclassified KD4-96 (order level; Figure 7B). In the PF, PSS had a higher abundance of MB-A2-108 and Thermoleophilia (class level) but a lower abundance in Flavobacteriales (order level) when compared with PSJ (Figure 7C). In the RCC, RSS had a greater abundance of Nitrospirota (phylum level), Acidobacteriae, Thermodesulfovibrionia, and Thermoleophilia (class level) but a lower abundance in Actinobacteriota (phylum level), Anaerolineae, and Actinobacteria (class level), as shown in Figure 7D.

### 3.5 Microbial function prediction

**Figure 8:**
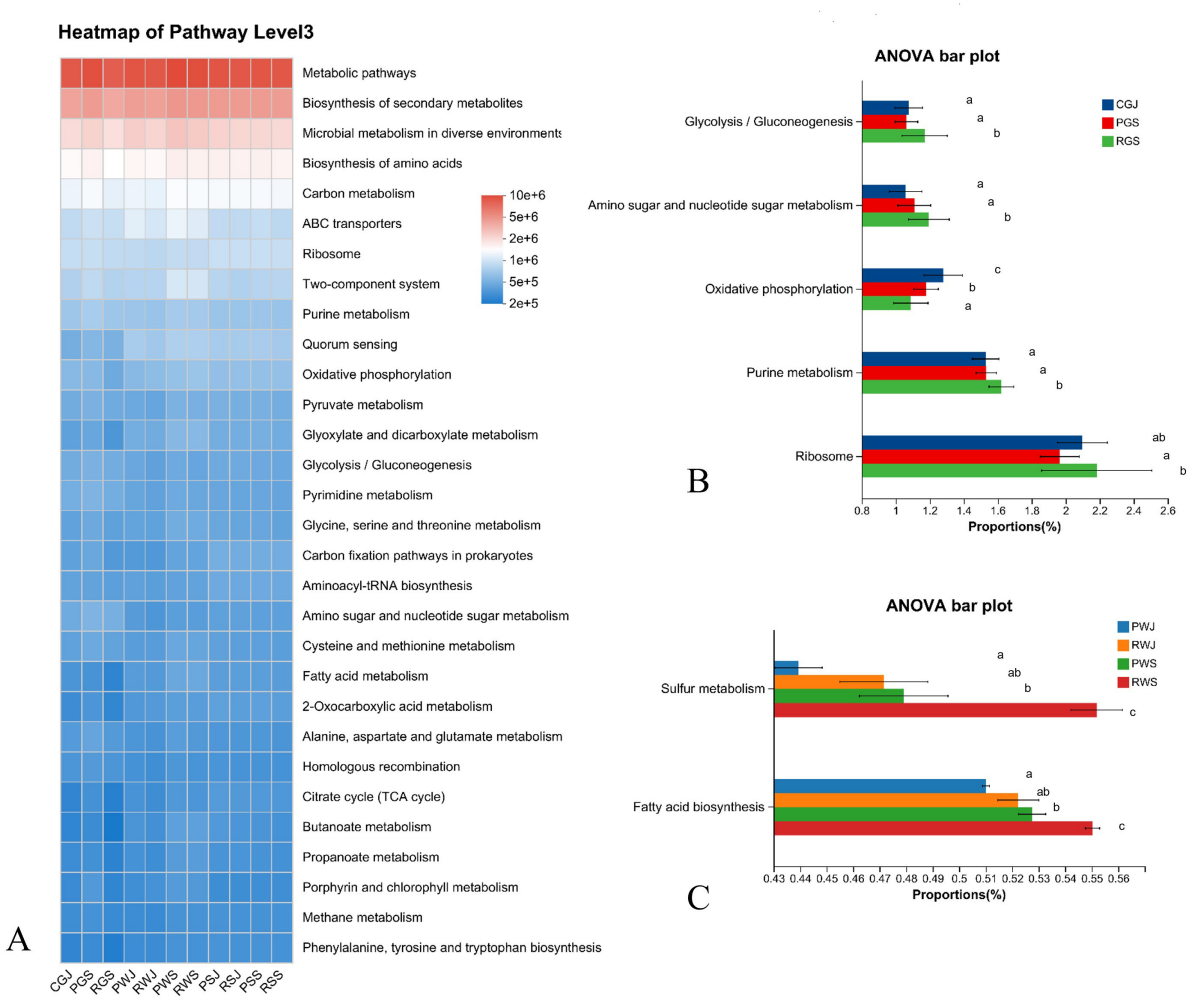
Predicted microbial functions (KEGG pathways, level 3) in gut, water, and sediment across two crab culture systems. **(A)** Heatmap of top 30 predicted functions, **(B)** Significant different pathways in gut samples (corrected *P* < 0.05), **(C)** Significant different pathways in water samples (corrected *P* < 0.05). *Different letters indicate* significant differences.

The relative abundance of predicted function pathways (top 30) based on the KEGG annotation at level 3 was shown in Figure 8A. The most abundant four KEGG pathways at level 3 were metabolic pathways, biosynthesis of secondary metabolites, microbial metabolism in diverse environments, and biosynthesis of amino acids. Among gut samples, significant difference was observed in Glycolysis/gluconeogenesis, Amino sugar and nucleotide metabolism, Oxidative phosphorylation, Purine metabolism, and Ribosome. Specifically, when compared with RGS, PGS had higher abundance in oxidative phosphorylation but lower abundance in the other four pathways (Figure 8B). As for water samples, only two pathways (Fatty acid biosynthesis and Sulfur metabolism) showed significantly different abundances and had the same pattens. RWS had the highest abundances, while PWJ had the lowest abundances in these two pathways (Figure 8C). In sediment samples, no significant different pathway was identified based on KEGG pathways.

**Figure 9:**
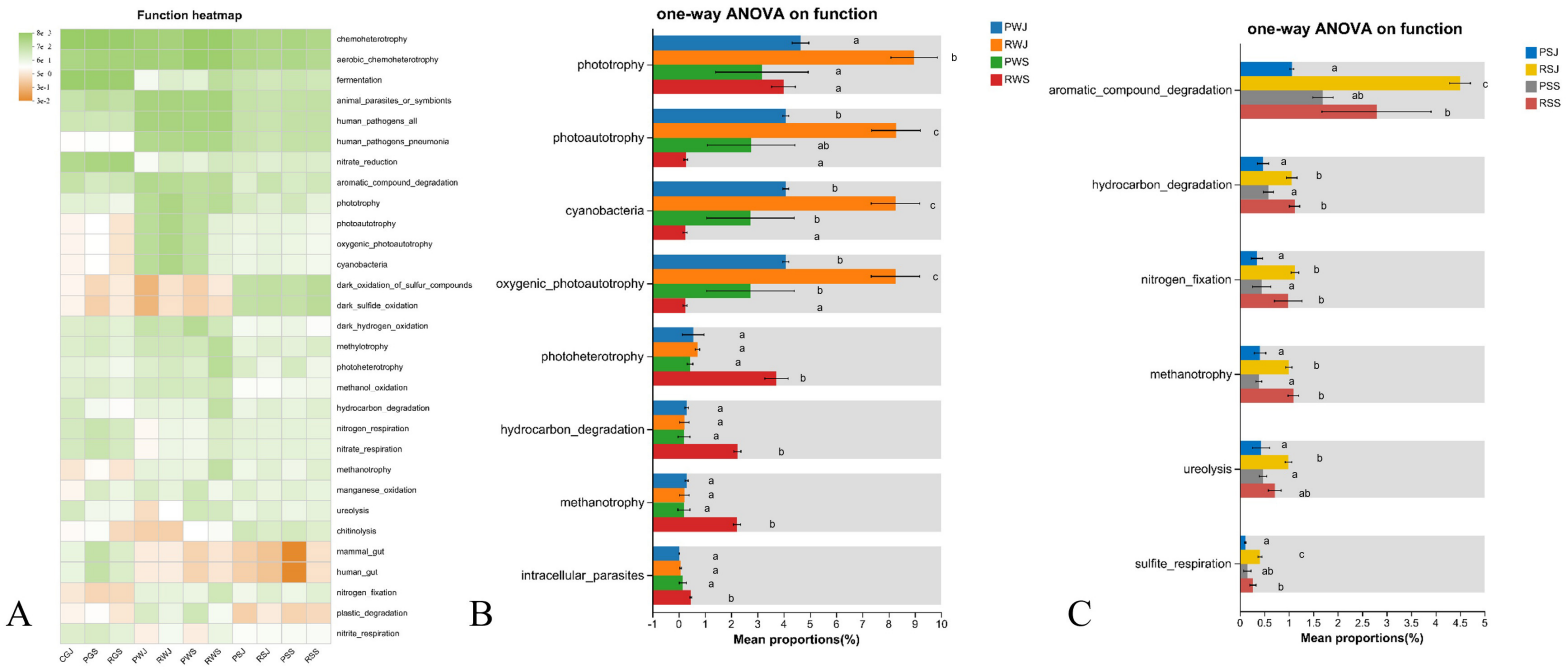
Predicted ecological relevant functions in microbial communities from water, and sediment across two crab culture systems. **(A)** Heatmap of top 30 predicted ecological relevant functions, **(B)** Significant different pathways among water samples (corrected *P* < 0.05), **(C)** Significant different pathways among sediment samples (corrected *P* < 0.05). *Different letters indicate* significant differences.

The ecologically relevant functions annotated by FAPROTAX are shown in Figure 9. In the water samples, functions related to Phototrophy, Photoautotrophy, Cyanbacteria, Oxygenic photoautotrophy, Photoheterotrophy, Hydrocarbon degradation, Methanotrophy, and Intracellular parasites showed significantly differences (Figure 9B). Generally, the abundances of the first four functions were the highest in RWJ. For Phototrophy, there were no significant differences in abundances among RWJ, PWS, and RWS. For the other three functions, the abundances in RWS were significantly lower than in RWJ and PWS. For the last four functions, the abundances in RWS were significantly higher than in PWJ, RWJ, and PWS, and the abundances among the latter three did not differ significantly. In comparisons among sediment samples, significant differences in abundance were observed in six functions, including Aromatic compound degradation, Hydrocarbon degradation, Nitrogen fixation, Methanotrophy, Ureolysis, and Sulfite respiration, as shown in Figure 9C. Overall, for all six functions, the abundances from samples of RCC (RSJ and RSS) were higher than those from PF (PSJ and PSS). In June, the abundances were significantly higher in RSJ compared to PSJ. In September, the abundances in RSS were higher than in PSS, although the differences were not significant for Aromatic compound degradation, Ureolysis, and Sulfite respiration.

### 3.6 Correlation analysis between gut and environmental microbiota

**Figure 10:**
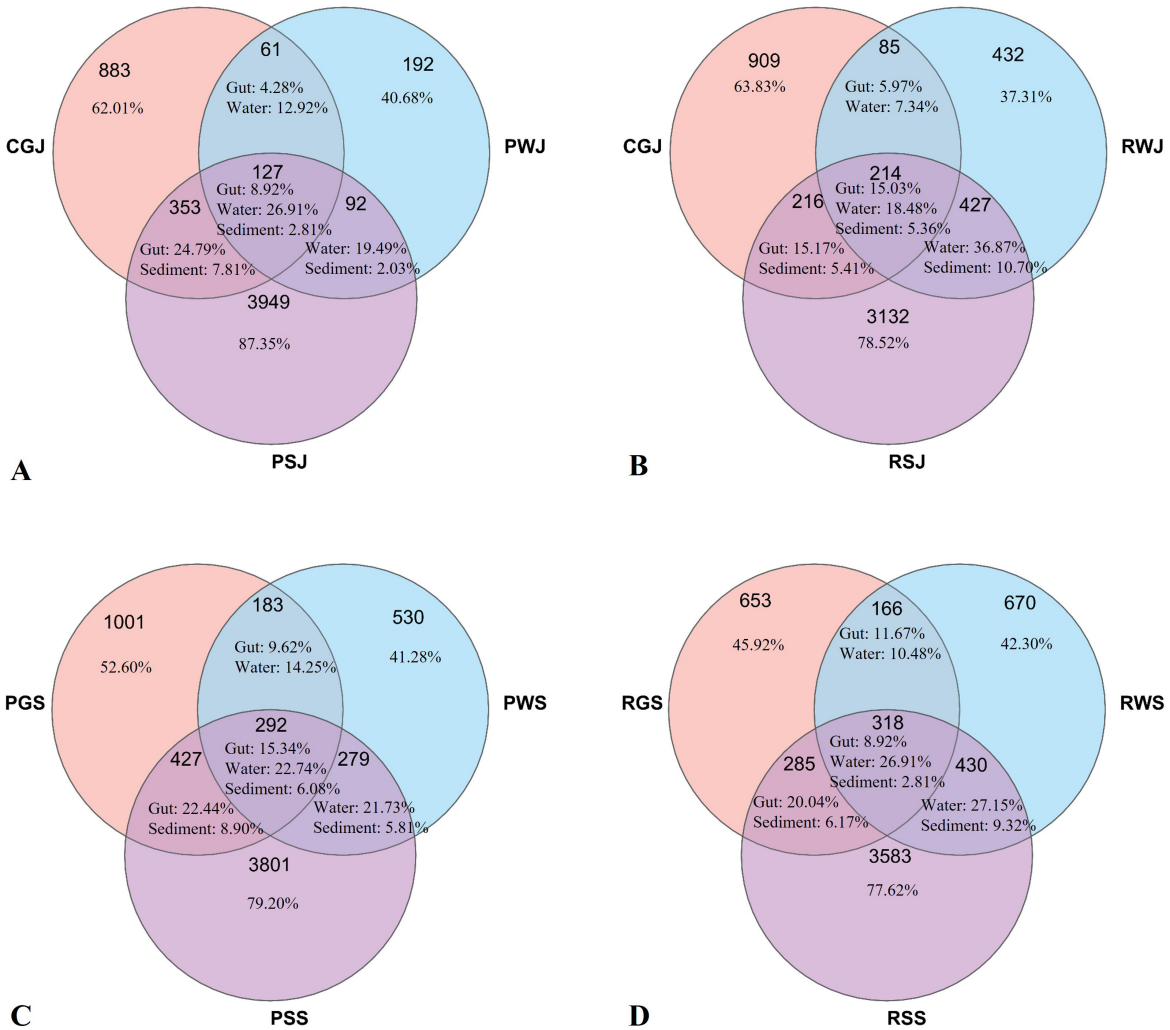
Venn diagram displaying the unique and shared OTU numbers among gut and environmental microbiota in two culture systems in June and September. **(A)** PF in June. **(B)** RCC in June. **(C)** PF in September. **(D)** RCC in September.

The Venn diagram exhibits the overlap of microbial OTUs among crab gut, water and sediment in June and September in two culture systems (Figure 10). In June, the gut unique OTUs accounted 62.01% and 63.83% in PGJ and RGJ, respectively. When the crabs were harvested in September, the amount of gut unique OTUs decreased to 52.60% and 45.92% in PGS and RGS, respectively.

## 4 Discussion

### 4.1 Differences in growth performance and enzymatic activities between two culture systems

In this study, the growth performance of crabs from the RCC was significantly better than crabs from the PF. In a previous study in tilapia (*Oreochromis niloticus*), better growth performance was also observed in fish from fish-rice coculture system compared to monoculture system (38), and the authors attribute it to superior water quality and additional nutrient acquisition in the fish-rice coculture system. In this study, before the release of the crabs, dissolved oxygen in the RCC was significantly higher than in the PF, while ammonia levels were significantly lower, as shown in the Supplementary Table S2. However, these differences were not significant in September. Chinese mitten crabs are omnivores, which have a highly diversified food resources including detritus, macrophytes, algae, fish, Arthropoda, Oligochaeta, Mollusca, and other aquatic invertebrates (39–41). Previous literature indicated that rice-animal coculture systems usually have higher biodiversity and more stable community structure (42, 43). For example, compared to rice monoculture system, the biodiversity of benthic and plankton fauna are more dynamic in rice-fish coculture system (44), and individual spider number is significantly greater in the rice-crab coculture system (45). Hu et al. investigated the dynamics of plankton structure in PF and RCC systems in Panjin City (46), which is the same region to our study. Significantly higher species richness, density, average biomass, and Shannon index in phytoplankton and zooplankton were observed in the RCC than PF. Aquatic animals in the rice-animal coculture system usually play a significant role in pest control (47). For example, common carps significantly reduce the abundance of stemboring moths, chironomid midges, and rice planthoppers in the rice fields (48, 49). Yellow finless eels (*Monopterus albus*) and loaches (*Misgurnus* spp.) significantly reduced the herbivore insect abundance by 24.07% (50). Due to its omnivorous behavior, the pest control of Chinese mitten crabs in rice fields is predictable, although the direct evidence is lack so far. Therefore, the higher biodiversity in the rice fields may provide crabs with extra nutrients, which contributes to the better growth performance of crabs from the RCC in this study.

The hepatopancreas of crustaceans is a key organ involved in digestion, detoxification, and immunity (51, 52). To assess the digestive and immunity capacities of crabs from two culture systems, we investigated the hepatopancreas' enzymatic activity of three digestive enzymes and two immune-related enzymes. Digestive enzymes represent a crucial link between the nutrients in the diet and those incorporated into the body for further use in basal metabolism and growth (53). In the present study, the levels of TRY and LPS in RS were significantly higher than that of PS, and the latter had similar levels to the CJ. Previous studies suggest that a moderate increase in protein and lipids in the diets contributes to higher levels of TRY and LPS in Chinese mitten crabs (54, 55). Therefore, the extra nutrition, probably animal-type food, in the RCC may contribute to this result. ACP and AKP are important hydrolases that play a significant role in digesting extracellular invaders and have a close relationship with phagocytosis (56, 57). They are considered sensitive immune parameters in crustaceans (58). In this study, higher ACP and AKP activities were observed in RS compared to PS. In the comparison of crayfish between rice-crayfish coculture and pond culture systems, ACP and AKP levels were higher in the former system (59), which aligns with our study. Therefore, crabs from RCC have higher levels of non-specific immune enzymes and are healthier compared to traditional PF.

### 4.2 α-diversity of gut and environmental microbiota

In each ecosystem, the highest α-diversity indices were observed in sediment microbiota compared to water and gut microbiota. The same results have been reported in previous literature across various aquaculture systems of crustacean species, including Chinese mitten crabs (23, 26), Kuruma prawns (*Penaeus japonicus*) (60, 61), tropical shrimp (*Penaeus notialis*) (62), and Pacific white shrimp (*Litopenaeus vannamei*) (63, 64). It is likely due to the abundant organic matter accumulated from aquaculture animal feces, food residuals, and other materials that serve as nutrients to support microorganism growth (61, 65).

### 4.3 Differences in gut microbiota between two systems

In the comparison between juvenile (June) and adult (September) crabs, PGS had significantly higher Chao index than CGJ and RGS, whereas no significant difference was observed between CGJ and RGS. As for Shannon indices, CGJ, PGS, and RGS did not differ significantly. Comparable Shannon indices between juvenile and adult aquaculture species have been reported in previous studies in Chinese mitten crabs (23), Pacific white shrimp (66), and common carps (*Cyprinus carpio*) (67).

Between the adult crabs from PF and RCC systems, although PGS and RGS owned similar Shannon indices, their β-diversity differed significantly. Specifically, compared with PGS, RGS was rich in Firmicutes (47.05% vs. 27.13%) but lower in Bacteroidota (22.38% vs. 34.34%). The gut microbiota of aquatic animals is evidently closely with their diets (68–70). In a comparison of the gut microbiota of Chinese mitten crabs fed plant- and animal-type diet, lower abundance of Bacteroidota and higher abundance of Firmicutes were observed in the group fed by animal-type diet when compared with plant-type diet (21). In this study, the same artificial diet was applied in ponds and rice fields. Therefore, as previously mentioned, the higher biodiversity in the rice fields may provide crabs with extra nutrients, especially the animal-type diet, which contributes to the lower abundance of Bacteroidota and higher abundance of Firmicutes in this study.

### 4.4 Differences in water microbiota between two systems

In the comparison of water microbiota between PF and RCC systems prior to the release of juvenile crabs in June, the abundance of cyanobacteria in RWJ (11.82%) was significantly higher than in PWJ (5.22%). This is probably due to fertilizer application in the rice field before transplanting the rice seedlings. By the harvest season in September, the abundance of cyanobacteria in RWS decreased to 0.39%, significantly lower than that in PWS (4.12%). Since the water was not released until the harvest season, the microbial community in September is more important compared to that in June. Cyanobacteria can potentially cause algal blooms and produce cyanotoxins, which is a significant concern for managing water quality in aquaculture systems (71, 72). A lower abundance of cyanobacteria in RCC indicated less impact on the environment after release. Order SAR11 bacteria (Alphaproteobacterial) also had a higher abundance in PWS compared with RWS, which plays a role in converting fixed carbon to atmospheric CO_2_ (73).

Due to the presence of rice, RWS is unsurprisingly rich in the Rhizobiales order compared to PWS. Members of the order Rhizobiales are beneficial in oxidizing nitrogen and methane (74, 75). Genera of *Rhizobiales Incertae Sedis* and *Methylocystis* had a higher abundance in RWS than PWS. The former can utilize a wide range of carbohydrates and is involved in nitrogen fixation (76, 77). *Methylocystis* spp. play an important role in reducing methane emission into the atmosphere from methanogenic wetlands (78). In addition to *Methylocystis* spp., the order Methylococcales also had higher abundance in RWS. Methylococcales members are aerobic methanotrophs–bacteria that can metabolize methane (79, 80). RWS also had higher abundance in *Arenimonas* spp. (gammaproteobacterial order Xanthomonadales), which can catalyze a variety of enzymes and play an essential role in the decomposition of organic matter. They enhance plant growth by improving the utilization rate of nutrients (81). The genus C39 (gammaproteobacterial family Rhodocyclaceae) had a higher abundance in RWS. Its high abundance has been detected in the water of plant and fish farming systems (82), herbivorous grass carp (*Ctenopharyngodon idella*) and carnivorous southern catfish (*Silurus meridionalis*) coculture systems (83), and rice–crayfish coculture systems (84). C39 possesses versatile metabolic capabilities, contributing to the removal of total nitrogen and total phosphorus (85).

### 4.5 Differences in sediment microbiota between two systems

In this study, significant differences in microbial community abundance were observed before release and during the harvest season. At phylum level, phyla of MBNT15 and Myxococcota had higher abundance in PF and RCC, respectively. MBNT15 bacteria are obligate anaerobes and usually thrive in anoxic sediment (86). In contrast, Myxococcota species are predominantly aerobic soil-dwelling microorganisms (87). The difference in the abundances of these two phyla between PF and RCC may reflect variations in the dissolved oxygen statuses of their sediments. In the Desulfobacterota phylum, species harbor sulfur-cycling bacteria (88). PF was rich in the Desulfuromonadia class, while RCC was rich in the Syntrophobacteria class. All members of the Desulfuromonadia class are mesophilic, whereas members of the Syntrophobacteria are either mesophilic or moderately thermophilic (89). The lower water depth in the rice field likely contributes to temperature increases in water and sediment, further affecting the abundance differences in Desulfuromonadia and Syntrophobacteria between the two systems.

Chloroflexi phylum had a high abundance in the sediment of PF and RCC, which is in line with previous studies (23, 90). Although the abundance of Chloroflexi did not differ significantly between two systems, higher abundance of KD4-96 class was observed in RCC than PF. KD4–96 species are proposed to be involved in C fixation by assimilating CO_2_ (91, 92). In the study on the rice-crayfish system, Huang et al. found that the relative abundance of Firmicutes phylum in the ponds without rice was significantly higher than that of the rice field (93), and relevant studies have confirmed that crops can significantly reduce the relative abundance of Firmicutes in sediment (94). In this study, the abundance of Firmicutes was slightly higher in PF compared with RCC, but not statistically significant. In Firmicutes, however, PF had a higher abundance of Bacilli class in PF compared to RCC. Some Bacillus species can colonize the rhizosphere or endophytic tissues of rice plants, benefiting rice cultivation by improving rice yield and resistance to diseases and wind (95, 96). Therefore, rice seeds or seedlings treated with *Bacillus* spp. can enhance germination, growth, and resistance to many pathogens (97–99). Whether the abundance of *Bacillus* spp. is sufficient in the rice fields in this study needs further research in the future.

Before the crab release in June, the abundance of the Nitrosomonadaceae family did not differ significantly between PF and RCC. However, by September, its abundance in RCC was significantly higher than in PF. Nitrosomonadaceae bacteria oxidize ammonia to nitrate (100), which is an important process in aquaculture systems (101) that helps maintain a better environment for Chinese mitten crabs. As expected, the abundance of the Rhizobiales order was higher in the RCC, as high abundance of Rhizobiales in the rice field has been reported by Kim and Liesack (102). Members of the Rhizobiales order play a beneficial role in nitrogen and methane oxidation, providing absorbable nutrients for plants and supporting sustain nutrient cycles within the field (75).

### 4.6 Microbial function potentials

Among gut samples, five metabolism-related KEGG pathways were significantly different in their abundance. For Glycolysis/gluconeogenesis, Amino sugar and nucleotide sugar metabolism, and Purine metabolism, abundances in RGS were significantly higher than in CGJ and PGS. A metagenomics analysis of gut microbiota of red swamp crayfish (*Procambarus clarkii*) revealed higher abundances of genes related to above-mentioned pathways in crayfish from rice fields compared to those from ponds (103). These results indicated that both red swamp crayfish and Chinese mitten crabs from the RCC could have better capabilities in those metabolism pathways. In this study, the abundance for Oxidative phosphorylation was lower in RGS than in CGJ and PGS. Although the increased function of oxidative phosphorylation has been associated with providing more free energy to host animal (104), it is also linked to higher redox potentials (105) and even a pathogenesis-related microbial function in Pacific white shrimp (106).

In terms of ecologically relevant functions in the water samples, the increases in Phototrophy, Photoautotrophy, and Oxygenic photoautotrophy in RWJ were attributed to its higher abundance in cyanobacteria, as previously mentioned. Compared to other samples, RWS had higher levels of Hydrocarbon degradation, Methanotrophy, and Intracellular parasites. Here, hydrocarbon mainly refers to methane, based on relative abundances. Methane, a potent greenhouse gas, is the second-largest contributor to climate warming after carbon dioxide (CO_2_). Both Chinese mitten crab aquaculture ponds and rice fields are significant contributors to atmospheric greenhouse gas (e.g., CO_2_, CH_4_, and N_2_O) (107, 108). Previous studies indicate that rice-animal coculture systems, such as rice-duck, rice-fish, and rice-frog can effectively alleviate greenhouse gas emissions compared to rice monoculture system (109). Although this advantage is less significant in the RCC (109), PF significantly increases the methane emission compared to rice monoculture field (108). In this study, the abundant methanotrophs (e.g., Rhizobiales, *Methylocystis* spp.) in RWS contributed to the higher methanotrophy function in RCC. This suggests that RCC system may reduce the methane emission compared with PF system, though a direct comparison is lacking.

In the sediment samples, similar to water samples, Hydrocarbon degradation and Methanotrophy functions were enhanced in RCC than in PF, which can be attributed to the higher abundance of Rhizobiales in the RCC. The high abundance of Rhizobiales also likely contributed to the increased function of Nitrogen fixation in RCC compared to PF. The function of Aromatic compound degradation was also increased in the RCC. Some aromatic compounds (e.g., benzoate, phenylpropionate and phenylacetate) are intermediates in the methanogenic degradation of rice straw and soil organic matter (110). Therefore, it is not surprising that sediment samples from the RCC had an increased capacity for this function. Similarly, urea fertilization in the rice field increased the Ureolysis function in RCC, and the increase in the abundance of soil ureolytic bacterial in the rice fields has been confirmed by previous study (111).

### 4.7 Correlation between gut and environmental microbiota

Previous studies on shrimp indicate that the gut microbiota of Kuruma prawns and Pacific white shrimp is closely associated with environmental microbiota (61, 64, 112). For example, the unique OTU number of gut microbiota of Kuruma prawns accounts for as low as 15.67% of the total gut OTU number. In this study, however, unique OTU numbers account for ~50% in either PF or RCC in September. This finding is similar to the previous studies on Chinese mitten crabs (26) and suggests that gut microbiota of crabs is not as closely related to the environment as that of shrimp. The reasons contributing to this difference between Chinese mitten crabs and Kuruma prawns and Pacific white shrimp require further investigation.

## 5 Conclusions

This study compared the growth performance, immune and digestive enzyme activities of crabs, as well as the microbiota in crab gut, water, and sediment between the RCC and PF systems. The results showed that RCC crabs exhibited better growth performance, and higher enzymatic activities of ACP and AKP, suggesting that RCC enhances the growth rate and immune response in Chinese mitten crabs. Significant differences were observed in the microbial communities of crab gut, water, and sediment samples between these two systems. RGS was characterized by an increased abundance of Firmicutes and a decreased abundance of Bacteroidota, suggesting that RCC crabs acquired additional nutrients, especially from animal-based diets, which was confirmed by their elevated LPS and TRY levels. In the surrounding environment, RCC was rich in various environmentally beneficial bacteria such as Rhizobiales, Methylococcales, KD4-96, C39, Xanthomonadales, and Nitrosomonadaceae. These bacteria play important roles in greenhouse gas reduction, carbon and nitrogen fixation, organic matter decomposition, and ammonia oxidation, respectively. The microbial function predictions also suggested that the water and sediment samples in the RCC had increased methanotrophy functions, with the sediment showing enhanced functions in nitrogen fixation, aromatic compound degradation. Conversely, PWS contained higher level of Cyanobacteria, which requires monitoring due to their potential to cause harmful algal blooms. Additionally, RCC sediment had a lower abundance of Bacillus species compared with sediment of PF, and these bacteria are considered beneficial for rice. Further investigation is needed to determine whether the abundance of *Bacillus* spp. is sufficient in the rice fields in this study.

This study deepens the understanding of the physiological status of crabs and expands our knowledge in RCC and PF ecosystems from the perspective of microbial communities. It provides insights into the advantages of RCC over traditional PF, particularly in promoting crab growth and improving environmental sustainability.

However, as this study was conducted in the Liaohe drainage during a single production cycle, other factors (e.g., location and climate) may influence microbial community. Long-term studies and research in different locations are needed to fully understand the microbiota dynamic in these two aquaculture systems. Additionally, we used *16S rRNA* sequencing in study, and metagenomic analyses in the subsequent studies can provide deeper insight into microbial functions.

## Data Availability

The datasets presented in this study can be found in online repositories. The names of the repository/repositories and accession number(s) can be found below: https://www.ncbi.nlm.nih.gov/, PRJNA1142732; https://www.ncbi.nlm.nih.gov/, PRJNA1142913; https://www.ncbi.nlm.nih.gov/, PRJNA1144156.
