# Supplementary material for: Comparative analysis of crab growth performance, enzyme activity, and microbiota between rice-crab coculture and pond farming systems
Source: Front Vet Sci. 2025 Mar 19;12:1571454. doi: 10.3389/fvets.2025.1571454 (PMC11961982; doi:10.3389/fvets.2025.1571454)
Supplement: Supplementary file 1 [file Table_1.docx]

Supplementary table1 Methods and assay kits for enzyme activity investigation

| **Name** | **Method** | **Catalogue number  of assay kit** | **Testing equipment** |
| --- | --- | --- | --- |
| TP | BCA method, Microplate method | A045-4-2 | ReadMax 1200 (Shanghai Flash Spectrum Biological Technology Co., Ltd., Shanghai, China) |
| ACP | Microplate method | A060-2-2 |  |
| AKP | Microplate method | A059-2-2 |  |
| LPS | Microplate method | A054-2-1 |  |
| AMS | Iodine-based colorimetry | C016-1-1 | T6 series UV-Vis spectrophotometer  (Persee Analytics Inc., Beijing, China) |
| TRY | Ultraviolet colorimetry | A080-2-1 |  |
