# Supplementary material for: Comparative analysis of crab growth performance, enzyme activity, and microbiota between rice-crab coculture and pond farming systems
Source: Front Vet Sci. 2025 Mar 19;12:1571454. doi: 10.3389/fvets.2025.1571454 (PMC11961982; doi:10.3389/fvets.2025.1571454)
Supplement: Supplementary file 2 [file Table_2.docx]

Supplementary table 2. pH, dissolved oxygen, and ammonia in two Chinese mitten crab culture system.

| Parameter | System | Month | |
| --- | --- | --- | --- |
|  |  | June | September |
| pH | PF | 8.613 ± 0.393 | 8.723 ± 0.336 |
|  | RCC | 8.663 ± 0.029 | 8.682 ± 0.332 |
| Dossolved oxygen  (mg/L) | PF | 5.850 ± 0.591^a^ | 4.267 ± 0.570 |
|  | RCC | 6.883 ± 0.159^b^ | 4.208 ± 0.313 |
| Ammonia (mg/L) | PF | 1.526 ± 0.154^b^ | 1.502 ± 0.167 |
|  | RCC | 1.204 ± 0.012^a^ | 1.303 ± 0.031 |

Different lowercase letters indicate significant differences between the two modes (*P* < 0 .05).
